# The Effect of Sex Discordance on Blood Transfusion Reactions

**DOI:** 10.30699/ijp.2021.534237.2683

**Published:** 2021-12-15

**Authors:** Mohammad Eini, Haniyeh Dadaie- Joushagani, Ebrahim Miri-Moghaddam

**Affiliations:** 1 Department of Hematology and Blood Banking, School of Paramedical Sciences, Birjand University of Medical Sciences, Birjand, Iran; 2 Student Research Committee, Department of Hematology and Blood Banking, School of Paramedical Sciences, Birjand University of Medical Sciences, Birjand, Iran; 3 Cardiovascular Diseases Research Center, Department of Molecular Medicine, Faculty of Medicine, Birjand University of Medical Sciences, Birjand, Iran

**Dear Editor**

According to a report released by the World Health Organization (WHO), about 118.5 million people around the world refer to the blood collection centers to donate blood annually, from whom only 32% (10-50%) were female donors in 2020 (1). In blood transfusion, the donated blood is tested for the infectious agents and the compatibility of blood groups. However, the gender mismatch is ignored, which can cause some complications. Most blood transfusion reactions are immunological rather than non-immunological (2). Blood donor sex can act as an effective factor in the development of immunological reactions at the time of injection for the following reasons:

1. Men who received blood from women with a history of pregnancy presented a higher reaction rate than those who received blood from women with no hisrtory of pregnancy . The presence of alloantibodies and transfusion-related acute lung injury would be the most important causes of reaction following blood transfusion (3, 4).

2. The male recipients' red blood cells (RBCs) are more susceptible to hemolysis during blood transfusion. Therefore, the level of free plasma hemoglobin (Hb) increases and causes haptoglobin depletion. Nitric acid accelerates the clearance of free hemoglobin by converting the Hb to Hi (methemoglobin). The excessive consumption of nitric oxide causes vascular dysfunction, increased platelet aggregation, and vascular damage. Older RBCs have less flexibility; hence they are trapped as they pass through the small vessels, thereby increasing the interaction of white blood cells (WBCs) and platelets with the blood vessels and leading to vascular damage (2).

3. RBCs express surface phosphatidylserine at an appropriate level for the activation of coagulation factors. By increasing RBC life span, phosphatidylserine is externalized to the cell outer membrane, providing a proper surface for the binding and activation of thrombin and fibrinogen due to the negative charge. The adhesive molecules on the RBCs attached to the wound site are not clear and need rephrasing before being activated. The presence of older RBCs in the men's bloodstream causes thrombosis and tissue damage (5).

4. During blood storage, phosphatidylserine is exposed on the outer membrane leaflet of RBCs, which causes immunomodulation and increase in the systemic inflammatory cytokine levels and tissue damage. These vesicles can also attach to the vascular endothelium and increase the levels of Von Willebrand Factor (VWF), E-selectin, and other adhesive molecules in the endothelial cells and activate the coagulation. Since older RBCs are higher in men than women, more extracellular vesicles are produced (5).

Unwanted blood transfusion reactions due to sex discordance may cause irreparable damages. There-fore, a more accurate understanding of these reactions and related biological processes. and prospective randomized trials may be helpful in prevention of the complications.

## Conflict of Interest

None.
